# Real-world cardiovascular effects of liraglutide: transportability analysis of the LEADER trial

**DOI:** 10.1101/2025.05.12.25327466

**Published:** 2025-05-30

**Authors:** Kevin Josey, Wenhui Liu, Theodore Warsavage, Morten Medici, Kajsa Kvist, Catherine G. Derington, Jane E.B. Reusch, Debashis Ghosh, Sridharan Raghavan

**Affiliations:** 1 US Department of Veterans Affairs Eastern Colorado Health Care System, Aurora, CO.; 2 Department of Biostatistics and Informatics, Colorado School of Public Health, Aurora, CO.; 3 Novo Nordisk A/S, Copenhagen, Denmark.; 4 Division of Cardiology, Department of Medicine, University of Colorado School of Medicine, Aurora, CO.; 5 Adult & Child Center for Outcomes Research & Delivery Science, University of Colorado Anschutz Medical Campus, Aurora, CO.; 6 Division of Diabetes, Endocrinology, and Metabolism, Department of Medicine, University of Colorado School of Medicine, Aurora, CO.; 7 Division of General Internal Medicine, Department of Medicine, University of Colorado School of Medicine, Aurora, CO.

## Abstract

**Objective:**

To evaluate generalizability of the Liraglutide Effect and Action in Diabetes: Evaluation of Cardiovascular Outcome Results (LEADER) randomized clinical trial (RCT) – a cardiovascular outcomes study of the glucagon-like peptide-1 receptor agonist (GLP-1RA) liraglutide – to US Veterans Affairs Healthcare System (VA) patients with diabetes, a population at high cardiovascular disease risk lacking direct RCT evidence of GLP-1RA efficacy.

**Design:**

Transportability analysis that integrates real-world and RCT data to estimate the average treatment effect of liraglutide versus placebo had LEADER enrolled VA diabetes patients.

**Setting:**

Multi-national RCT and US VA

**Participants:**

9,336 LEADER participants and 357,075 VA users with diabetes from 2015–2023

**Interventions:**

Liraglutide versus placebo

**Main Outcomes and Measures:**

Risk differences (RD) in survival probabilities and hazard ratios (HR) of trial-adjudicated major adverse cardiovascular events (MACE) (composite of non-fatal myocardial infarction, non-fatal stroke, and cardiovascular mortality) and all-cause mortality, estimated with augmented inverse probability weighting after balancing baseline characteristics between LEADER participants and trial-eligible veterans using approximate balancing weights. Sensitivity analyses varied VA cohort composition and balancing variables.

**Results:**

Transported effects of liraglutide compared to placebo on MACE and all-cause mortality in veterans (“VA-weighted LEADER”) consistently overlapped the treatment effects observed in LEADER: RD of 3-year MACE of 2.0% [95% CI 0.8, 3.2] in VA-weighted LEADER versus 1.6% [0.3, 2.9] in LEADER; MACE HR 0.74 [0.61, 0.90] in VA-weighted LEADER versus 0.87 [0.78, 0.97] in LEADER; RD of 3-year all-cause mortality of 1.5% [0.6, 2.4] in VA-weighted LEADER versus 0.9% [−0.09, 1.9] in LEADER; all-cause mortality HR 0.71 [0.57, 0.89] in VA-weighted LEADER versus 0.85 [0.74, 0.97] in LEADER. Results were robust to all sensitivity analyses.

**Conclusions:**

The benefits of liraglutide observed in LEADER generalized to veterans with diabetes - real-world evidence that can guide diabetes treatment decisions for a high-risk population underrepresented in RCTs and inform formulary policies for an integrated healthcare system.

## Introduction

Randomized clinical trials (RCTs) provide gold-standard evidence for evaluating medication efficacy but often have uncertain external validity, especially when RCT participants are not representative of potential real-world users of a medication.^1^ This uncertain external validity can complicate treatment decision-making at the point of care and may underlie delays in uptake of new, effective therapies.^1,2^ Furthermore, RCTs often motivate regulatory decisions, treatment guidelines, and medication access policies, particularly for new therapies, resulting in regulatory approval, guidelines, and policies that impact diverse real-world populations based on data from non-representative RCT samples.^3–5^ The disconnect between individuals sampled into RCTs and real-world patient populations is particularly acute in type 2 diabetes, a highly prevalent condition affecting a heterogeneous population with a substantial expansion in treatment options over the last decade. For example, RCTs of glucagon-like peptide-1 receptor agonists (GLP-1RAs), potent agents for the treatment of type 2 diabetes and obesity^6,7^ with demonstrated cardiovascular benefits,^6^ have consistently under-enrolled relevant constituencies of potential medication users, leading to gaps in our understanding about the effectiveness of these drugs in many populations.^8–13^ Moreover, many newer diabetes medications with cardiovascular benefits, including GLP-1RA, are expensive, making their use and accessibility sensitive to treatment coverage policies.

While the most direct method for assessing medication effects in specific target populations is to conduct an RCT within each population, this strategy is impractical, and often infeasible, due to high costs and extensive time requirements. Observational studies using real-world data, collected after a medication is in routine use, offer an appealing alternative to RCTs but can be limited by confounding and sampling bias. To address these limitations, transportability analyses have emerged as an alternative, middle-ground solution.^14,15^ Methods for transporting effect estimates integrate RCT and real-world data to estimate what the treatment effect would have been if individuals from the real-world target population had been enrolled in the trial.^16^ An important feature of these methods is that they preserve the benefits of randomization (i.e., minimizing confounding) and can utilize data on potential users of a medication without requiring real-world uptake, limiting selection bias and delays in real-world evidence generation. Thus, transportability analyses can contribute evidence of efficacy for new medications in representative populations and inform formulary policies for healthcare payors servicing real-world potential users of a new medication.

The objective of this study was to assess external validity of the Liraglutide Effect and Action in Diabetes: Evaluation of Cardiovascular Outcome Results (LEADER) study – the first RCT to demonstrate cardiovascular benefits of a GLP-1RA (liraglutide)^17^ – in a real-world population under-represented in trials. We transported the results of the LEADER study^17^ to US Department of Veterans Healthcare System (VA) users with type 2 diabetes, a real-world patient population comprising a small minority in foundational RCTs of GLP-1RAs^6,17–19^ despite having higher cardiovascular disease risk than non-veteran US adults with diabetes.^20,21^ The study provides real-world evidence to guide VA formulary policies for GLP-1RA in a high-risk population in which direct RCT data are lacking.

## Methods

### Data sources

Individual-participant data from the LEADER study was shared with the study team through a data use agreement with Novo Nordisk A/S. All VA data were derived from the Corporate Data Warehouse, which records diagnoses, labs, and medication use for individuals receiving care at the VA. Veterans with type 2 diabetes were identified using a validated algorithm for the VA based on the presence of two or more ICD-9 or ICD-10 diagnosis codes for diabetes within a two-year period starting from January 1, 2002.^22,23^ To ensure the study population demonstrated evidence of active VA care, we required participants to have at least two outpatient encounters including at least one VA primary care provider visit and at least one prescription medication filled through the VA within two years of the first occurrence of a diabetes diagnosis code. The VA Eastern Colorado Health Care System Research & Development Committee and the Colorado Multiple Institutional Review Board provided human subjects review and approval of the study.

#### Target populations

The target population is represented by a cohort of veterans with type 2 diabetes receiving care in the VA between 2015 and 2023, corresponding to years after the initial results of the LEADER trial were first publicly reported. We initially translate the major inclusion and exclusion criteria of the LEADER trial onto the VA data to create a LEADER-eligible VA cohort, as shown in eTable 1. As in LEADER, VA patients could previously or contemporaneously be untreated for diabetes or treated with oral hypoglycemic medications, long-acting insulin, or a combination of these agents. Unlike the LEADER study, however, we excluded individuals with estimated glomerular filtration rate (eGFR) calculated with the Modification of Diet in Renal Disease^24^ equation <15 ml/min/1.73m^2^. From these administrative criteria, the primary VA target population (“Cohort A”) most closely matches the LEADER study sample, requiring individuals to have a history of atherosclerotic cardiovascular disease (ASCVD), age ≥50 years, and HbA1c ≥ 7% at baseline. LEADER included individuals with age ≥60 years with at least one cardiovascular risk factor, but this criterion was not mapped to the VA due to uncertainty surrounding the data quality for the cardiovascular risk factors used to define LEADER eligibility.

To examine the potential benefit of liraglutide use in pragmatic populations that were not trial-eligible, additional VA target populations were created for sensitivity analyses, relaxing three key inclusion criteria of LEADER: Cohort B relaxed the HbA1c requirement; Cohort C removed the age restriction; Cohort D did not require a history of ASCVD; and Cohort E imposed none of these three criteria.

#### Intervention

The real-world treatment in the target population is not used in the analysis, as the transportability method estimates the effect of the randomized LEADER interventions in the VA population. The intervention of interest was the LEADER RCT intervention: liraglutide (1.8 mg daily or maximum tolerated dose) or placebo.^17^

#### Outcomes

All outcomes are derived from the LEADER data; real-world outcomes in the target population are not used for transportability analyses. The primary endpoint was time to first major adverse cardiovascular event (MACE), a composite of cardiovascular death, non-fatal myocardial infarction (MI), or non-fatal stroke.^17^ Time to non-fatal MI, non-fatal stroke, and all-cause mortality were secondary outcomes. We also evaluated the incidence of selected adverse events reported in LEADER: all-cause medication discontinuation, gastrointestinal events (treatment-interrupting severe nausea/vomiting/diarrhea), pancreatitis, a gallbladder-related composite (acute gallstone disease, cholelithiasis, or cholecystitis), and any incident malignant neoplasm.

#### Covariates

We identified potential effect modifiers and confounders of trial participation – variables that might differ between the trial and VA populations which also may modify treatment effects: age, self-reported race, measures of diabetes severity (HbA1c, diabetes duration, number of diabetes medications), comorbidities (history of cardiovascular conditions; chronic kidney disease stage; atrial fibrillation; chronic obstructive pulmonary disease [COPD]; cancer; liver disease; dementia), and other risk factors (body mass index [BMI], blood pressure, lipids, kidney function, smoking status).^23,25^ We did not include sex as a covariate in the primary analysis due to the VA sample being predominantly men, though we did include sex in a sensitivity analysis.

#### Analytical approach

The conceptualization and biostatistical methods underlying the transportability analysis are included in the Online Supplemental Methods. We first computed pseudo-observations^26^ for individual survival probabilities of MACE at 6-month intervals from 6 to 54 months. By implementing this pre-processing step, we can treat the survival pseudo-observations as quasi-continuous outcomes so that we may apply standard causal inference methods without having to fit complex censoring-adjusted time-to-event models.^27^

We constructed doubly-robust estimators to estimate treatment effects specific to the target population by transporting average treatment effects from the RCT to the VA target population.^14^ To adjust for differences between the RCT and VA target population, we derived approximate balancing weights for each trial participant, which quantify their representativeness of the VA population. These weights “approximately balance” the covariates as they include a penalty term which ensures that the weighted covariate averages from the RCT match the target population within a small tolerance.^28,29^ We verified covariate balance between the weighted trial sample and the target VA sample by examining standardized mean differences (SMDs) for all covariates considered to be potential effect modifiers.^30^ In addition to the weighting adjustment, we fit an outcome regression model using Super Learner on the trial data to predict survival probabilities as a flexible ensemble method relating the treatment assignment and participant covariates to the outcomes.^31^ The final treatment effect estimates for the VA target population were computed by combining the weighting and outcome regression models into an augmented inverse probability weighted estimator.^14,16,32^ This estimator yields consistent treatment effect estimates f either the outcome or the weighting model is correctly specified.^33^

For each outcome, we computed the risk difference (RD) in event-free survival between liraglutide and placebo in a target population, along with the corresponding 95% confidence intervals (CIs) (details on estimation of standard errors can be found in the Online Supplemental Methods). Additionally, we calculated hazard ratios (HR) by fitting weighted Cox proportional hazards models on the LEADER trial data, applying the same weights used to estimate survival differences.^34^ These Cox models adjusted for effect modification through only the weights without coupling an outcome regression model. Consequently, unlike the RD estimates, the HR estimates are not doubly robust.

Several sensitivity analyses were conducted to verify robustness. First, we explicitly included sex as one of the balancing covariates. Second, we performed a leave-one-out analysis to determine whether excluding any single covariate from the set of covariates we balance and adjust in our weighting and outcome models significantly impacted the transported treatment effect estimates. Third, to assess sensitivity to the definition of the target population, we repeated these analyses for alternative cohorts (Cohorts B through E), with particular focus towards populations that include veterans currently ineligible for GLP-1 receptor agonist (GLP-1RA) treatment under existing VA formulary policies.

All analyses were completed in R v4.4.1 (R Core Team 2025, Vienna, Austria).

#### Patient and public involvement

The study was a secondary analysis of already collected data. No patients or other members of the public participated in developing the research question, the design of the study, or the reporting of the results.

## Results

### Baseline Characteristics

Cohort A (LEADER-eligible VA cohort) included 357,075 veterans meeting LEADER’s inclusion and exclusion criteria, compared to 9,336 participants in the LEADER trial. Table 1 summarizes baseline characteristics of these two groups. Veterans were older on average (mean age of 70.0 years versus 64.3 years in LEADER), predominantly male (97.5% vs. 64.3% in LEADER), and were more likely to be Black or African American (14.1% vs. 8.3%). Baseline BMI (approximately 32.5 kg/m^2^) and HbA1c (8.8% vs. 8.7%) were similar between VA and LEADER samples. Veterans had markedly higher rates of heart failure (21.1% vs. 14.0%), atrial fibrillation (16.3% vs. 1.9%), COPD (22.3% vs. 1.4%), and cancer (36.8% vs. 5.9%) compared to LEADER participants. Veterans also had worse baseline kidney function (mean eGFR 66.4 vs. 79.1 ml/min/1.73m^2^) and higher rates of current or former smoking (77.7% vs. 58.6%). After applying sampling weights, SMDs between the LEADER and VA samples were reduced to <0.05 for all balancing variables (eFigure 1).

#### Outcomes

**Figure 1.**
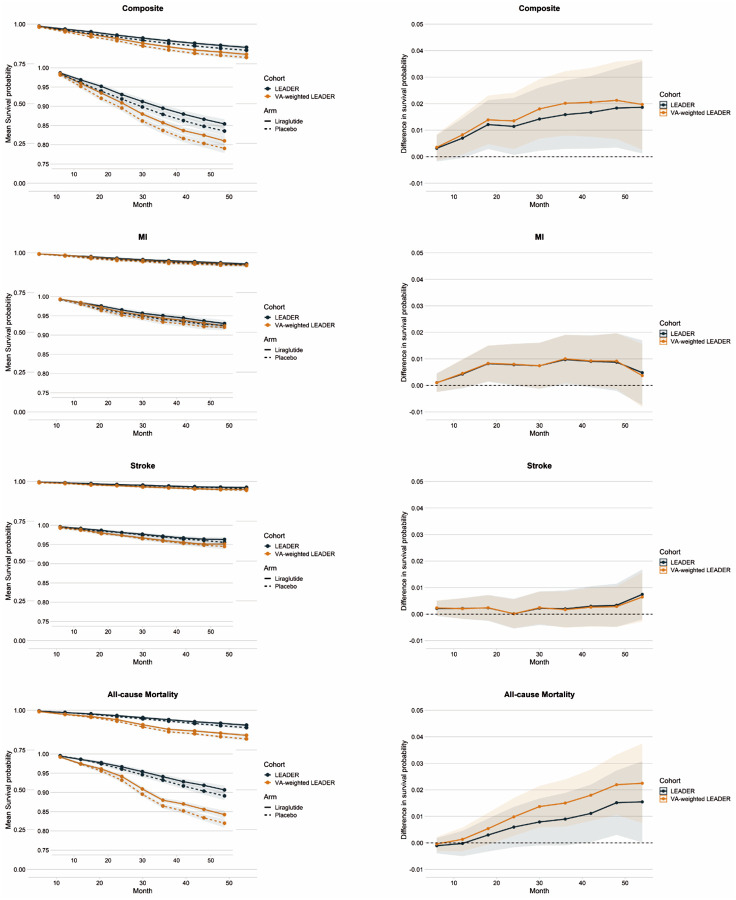
(**Left panel**) Predicted survival probabilities in liraglutide (solid lines) and placebo (dashed lines) arms of LEADER trial (blue) and transported to VA target population (yellow) for composite major adverse cardiovascular events (Composite), non-fatal myocardial infarction (MI), non-fatal stroke, and all-cause mortality. (**Right panel**) Absolute risk differences in event-free survival (liraglutide minus placebo) for composite major adverse cardiovascular events (Composite), non-fatal myocardial infarction (MI), non-fatal stroke, and all-cause mortality in LEADER (blue) and transported to VA target population (yellow); 95% confidence intervals are shown with colored shading.

The transported survival probabilities for the primary MACE outcome and secondary outcomes in the primary VA target population were lower for both the liraglutide and placebo arms than in the original LEADER study (Figure 1A). Randomization to liraglutide was associated with a risk reduction of the primary MACE composite outcome and each secondary outcome compared to placebo. At 36 months of follow-up, the RD in outcome-free survival between the liraglutide and placebo arms was: 2.01% (95% CI: 0.80%, 3.22%), favoring liraglutide, for the composite MACE outcome in VA-weighted LEADER and 1.59% (0.30%, 3.04%) in LEADER; 0.17% (−0.05%, 0.85%) for stroke in VA-weighted LEADER and 0.20% (−0.49%, 0.90%) in LEADER; 1.00% (0.09%, 1.91%) for MI in VA-weighted LEADER and 0.97% (0.04%, 1.90%) in LEADER; and 1.50% (0.62%, 2.39%) for all-cause mortality in VA-weighted LEADER and 0.90% (−0.09%, 1.89%) in LEADER (Figure 1B). For all outcomes and at all follow-up time points, the transported RD in the VA target population fell within the 95% confidence interval of the corresponding treatment effect in LEADER (Figure 1B). The RDs of the composite MACE outcome and all-cause mortality, but not for stroke and MI, were qualitatively greater when LEADER was transported to the VA target population.

**Figure 2.**
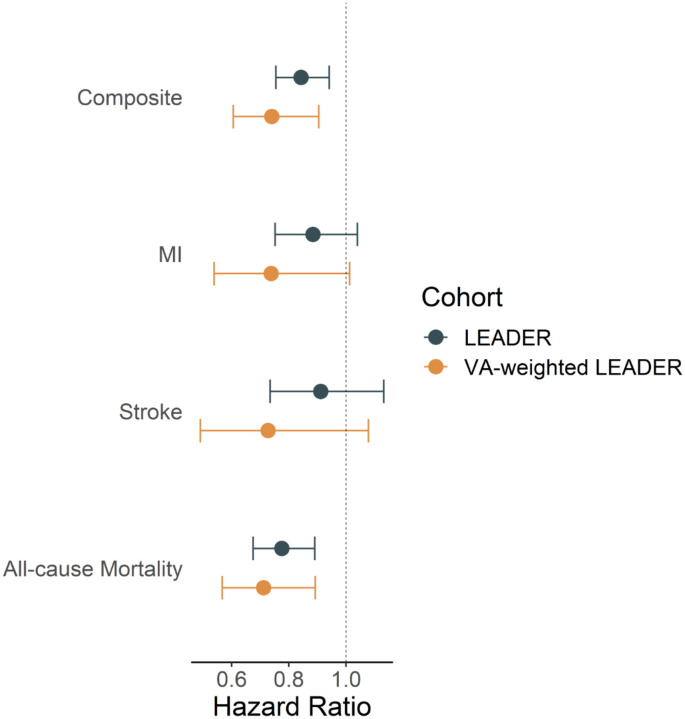
Hazard ratios and 95% confidence intervals estimated in LEADER (blue) and transported to VA target population (yellow) comparing randomized treatment to liraglutide relative to placebo for composite major adverse cardiovascular events (Composite), non-fatal myocardial infarction (MI), non-fatal stroke, and all-cause mortality. Hazard ratios less than 1 (vertical dotted line) favor liraglutide.

The transported HR for MACE in VA-weighted LEADER was 0.76 (0.62, 0.94) for liraglutide versus placebo, which was a somewhat larger relative effect than observed in LEADER (HR 0.87 [0.78–0.97]) (Figure 2). A similar pattern was observed for each of the secondary outcomes: the CIs overlap between the relative effects estimated in LEADER and in the VA target population (Figure 2).

#### Adverse Events

**Figure 3.**
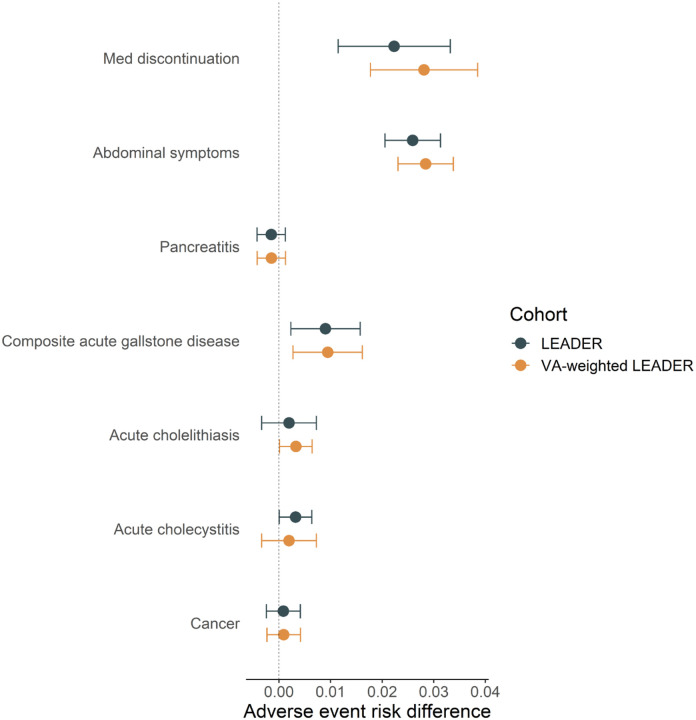
Absolute risk differences at the end of follow-up and 95% confidence intervals for several adverse events recorded in the LEADER trial (blue) and transported to the VA target population (yellow). Risk differences greater than 0 (vertical dotted line) reflect greater risk of adverse events in individuals randomized to liraglutide compared to placebo.

To assess external validity in the VA target population of safety outcomes assessed in LEADER, we estimated transported effects of liraglutide versus placebo on selected adverse events. As with the primary and secondary efficacy outcomes, the transported RDs for all adverse events examined fell within the 95% confidence intervals estimated in LEADER (Figure 3). For several of the adverse events examined, we observed a qualitatively greater risk associated with randomization to liraglutide versus placebo in VA-weighted LEADER than in LEADER (Figure 3).

#### Sensitivity Analyses

**Figure 4.**
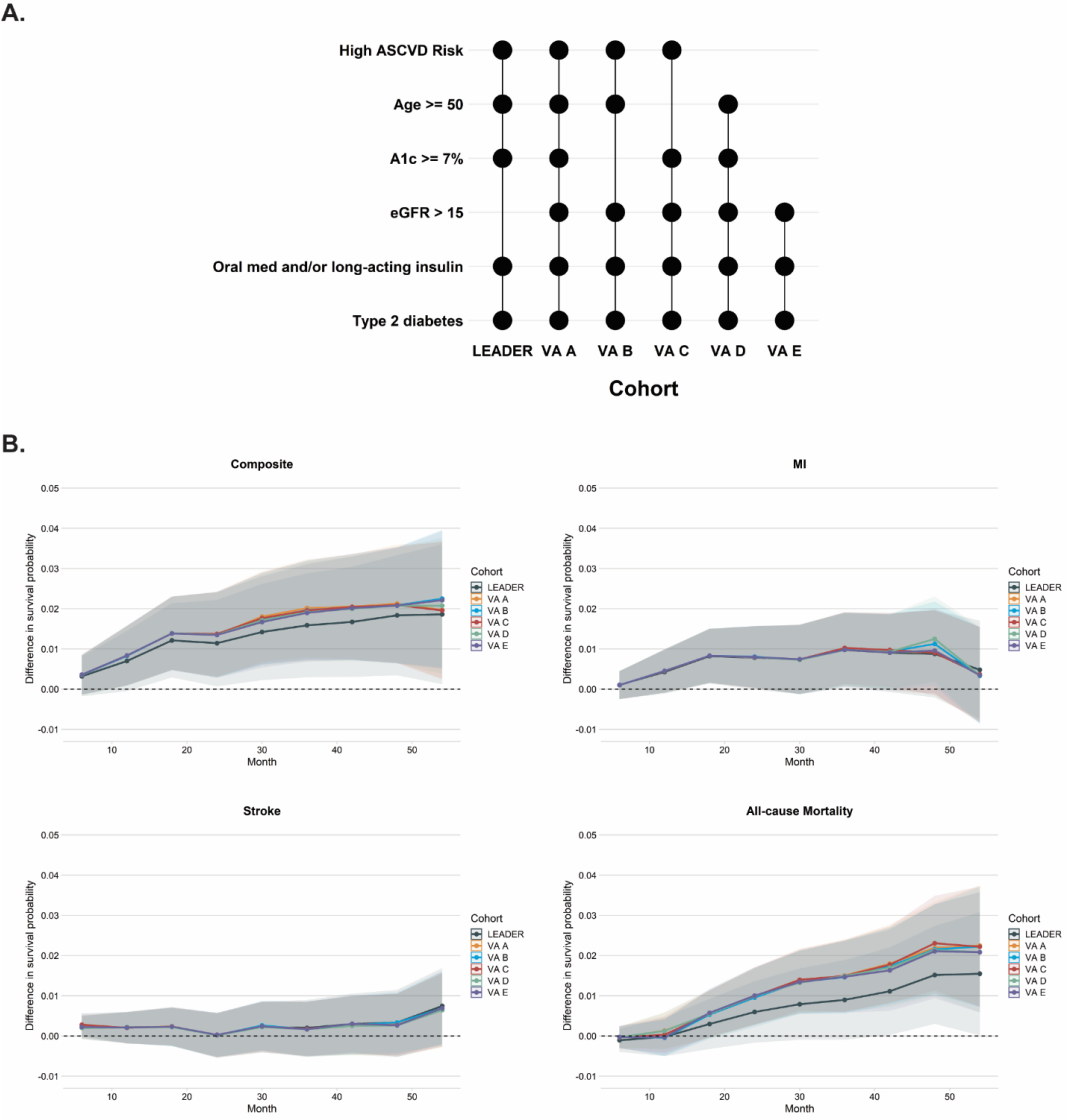
Comparison of treatment effects in LEADER and LEADER transported to a series of VA target populations with varying inclusion/exclusion criteria. (**A**) Key inclusion criteria for LEADER and for each VA target population included in the analysis. (**B**) Transported treatment effect estimated as survival probability differences for composite major adverse cardiovascular events (Composite), non-fatal myocardial infarction (MI), non-fatal stroke, and all-cause mortality; 95% confidence intervals are shown with colored shading for each transported treatment effect estimate. VA A through VA E indicate results after weighting LEADER to the corresponding target population. Abbreviations: ASCVD, atherosclerotic cardiovascular disease; A1c, hemoglobin A1c; eGFR, estimated glomerular filtration rate calculated using the Modification of Diet in Renal Disease equation (units of ml/min/1.73m^2^).

The transported RDs estimated for the VA target population were nearly identical without and with sex as a balancing variable (eFigure 2). In the leave-one-out-analysis, baseline age, HbA1c, and number of diabetes medications qualitatively impacted the transported effect estimates on the absolute (RD) and relative (HR) scales for the primary and secondary outcomes (eFigure 3). However, regardless of the balancing variable omitted, the transported effects in the VA target population for all outcomes on the RD and HR scales remained within the 95% confidence intervals of the LEADER study treatment effect estimates. Finally, the absolute RD and HR of the primary and secondary efficacy outcomes was similar across all VA target populations – within the 95% confidence intervals of the LEADER study with qualitatively greater absolute and relative risk reduction for the composite MACE and all-cause mortality (Figure 4 and eFigure 4).

## Discussion

Transporting treatment effects across populations, we found that the LEADER trial examining cardiovascular outcomes associated with liraglutide generalized to a real-world cohort of US veterans with type 2 diabetes. Liraglutide was associated with reduced risk of the composite MACE outcome and all-cause mortality in a largely male, elderly VA target population. While the transported effect estimates for all outcomes fell within the 95% confidence intervals estimated in the LEADER study, the modestly greater RD for the primary MACE outcome and all-cause mortality implies the number needed to treat (NNT) to prevent one MACE event or one death over three years may be smaller in veterans than in the LEADER population (NNT of 50 versus 62.5 for MACE and 67 versus 111 for all-cause mortality). These lower NNTs in veterans suggest that treating 12–44 fewer patients would be needed to prevent one MACE or mortality event, respectively, compared to the LEADER population, reflecting the higher baseline risk and potentially greater absolute benefit in this population. Moreover, the sensitivity analysis in broader VA target populations suggests liraglutide may confer benefit outside the trial inclusion/exclusion parameters and outside the current criteria for use of GLP-1RA in the VA. This reinforces the cardioprotective role of liraglutide as previously demonstrated in RCTs like LEADER, and importantly, extends that evidence to a broader population of potential users.

Our findings extend prior studies that replicate or generalize GLP-1RA cardiovascular outcome trials with real-world data in a variety of settings. The sensitivity analysis results in Cohorts D and E demonstrating cardiovascular benefit of liraglutide in veterans irrespective of pre-existing ASCVD mirror real-world studies evaluating GLP-1RA for ASCVD primary prevention and subgroup analyses in GLP-1RA RCTs that did not demonstrate heterogeneity by ASCVD history.^6,35^ Our results also support a recent systematic review that found concordance between RCTs and real-world studies for GLP-1RAs^36^ despite real-world patients with diabetes infrequently meeting eligibility criteria for GLP-1RA RCTs.^11^ Importantly, transportability analyses explicitly combine RCT data with real-world data unlike prior secondary analyses of RCTs or studies exclusively using real-world data, leveraging the internal validity of the RCT while extending inferences to the target population.

Transportability analyses may help inform treatment policies when constituents of a specific healthcare system are not well-represented in an RCT, and we demonstrate this using data from the largest integrated healthcare system in the US. In the VA, formulary decisions and criteria for use are often based on demonstrated outcomes in veterans. Our study provides evidence that can inform those decisions in the absence of VA-based RCTs.^37,38^ The primary analyses support the use of GLP-1RAs for patients with type 2 diabetes and established ASCVD or high risk of ASCVD. The sensitivity analyses in broader VA target populations suggest that several of the key LEADER inclusion criteria were inessential for liraglutide to be beneficial in veterans. As several of the RCT inclusion criteria – e.g., prior ASCVD and elevated A1c – remain among the criteria for use of GLP-1RA in the VA, the results with more broadly defined target populations argue for further study of expanding access to these agents balanced against cost, clinical characteristics, patient preferences, and drug availability. With the recent and ongoing growth in new therapies for cardiometabolic conditions, analogous analyses can guide evaluation of external validity of new treatments in populations relevant to policy-makers, payors, and healthcare systems. Critically, transportability analyses can be performed with real-world target population data for potential users of a new medication before treatment uptake in routine care, allowing rapid, time-sensitive real-world evidence generation to inform policies regarding new therapies.

Our study has important limitations. First, transportability assumes that any censoring is non-informative or that the censoring mechanisms would be similar in the target population and in the RCT. Second, while we included many covariates for adjustment, unmeasured differences or effect modifiers between the trial and VA populations could exist. For example, psychosocial factors, diet, exercise, adherence behaviors, or quality of background care might differ and were not captured. However, leave-one-out sensitivity analysis demonstrated that no single effect modifier included in the analysis meaningfully impacted the transported effect estimate, suggesting that the impact of an unmeasured effect modifier would have to substantially exceed the impact of any of the measured factors examined in the study. Third, the VA population is predominantly older men with extensive comorbidities. While excess disease burden in veterans helps motivate our study, it also means that the external validity of our conclusions is strong for VA and similar target populations, but caution is needed in extrapolating to populations with different characteristics. Finally, even with a very large VA sample, the statistical power for transported estimates is governed by the trial size and the variation in weights. We found that the effective sample size of LEADER that provides information relevant to the primary VA target population was only 2477 out of the 9336 participants in the LEADER study. This means that despite the large LEADER trial, only about one-quarter of participants were similar enough to veterans to contribute meaningful information about treatment effects in the VA population. This effective sample size declined further when examining broader veteran populations in sensitivity analyses (eFigure 5). The effective sample size estimates highlight the limited statistical power to detect significant differences between LEADER and transported effects, though that was not the goal of the study. The resulting wide confidence intervals reflect that transporting an effect is not as precise as conducting an RCT in the target population.

By transporting the LEADER trial results to a real-world population, we found that liraglutide’s cardiovascular and mortality benefits generalize to US adults with type 2 diabetes receiving care in the VA, the largest integrated healthcare system in the US. The results support the use of liraglutide to mitigate cardiovascular disease risk in a real-world population that was poorly represented in the RCT. The study also illustrates the value of causal transportability analyses in bridging the gap between clinical trial efficacy and real-world effectiveness. As healthcare systems increasingly rely on real-world evidence to inform practice and policy, the approach applied here – leveraging robust RCT results and tailoring them to a target population – can serve as a model for evaluating external validity of RCTs of other new therapies to inform medication access policies.

## Supplementary Material

Supplement 1

## Figures and Tables

**Table 1.** Study participant characteristics at baseline.

	VA	LEADER	p-value
Total N	357,075	9,336	
Male sex, n (%)	348,101 (97.5)	6,001 (64.3)	<0.001
Race, n (%)			<0.001
Black or African-American	50,312 (14.1)	776 (8.3)	
White	279,341 (78.2)	7,237 (77.5)	
Other	27,422 (7.7)	1,323 (14.2)	
Age (years), mean (SD)	70.0 (8.7)	64.3 (7.2)	<0.001
Hemoglobin A1c (%), mean (SD)	8.8 (1.7)	8.7 (1.5)	<0.001
# of diabetes medications, n (%)			<0.001
≤1	173,632 (48.6)	1,313 (14.1)	
2	87,554 (24.5)	3,294 (35.3)	
≥3	95,889 (26.9)	4,729 (50.7)	
BMI (kg/m^2^), mean (SD)	32.6 (6.4)	32.5 (6.3)	0.466
eGFR (ml/min/1.73m^2^), mean (SD)	66.4 (21.4)	79.1 (22.1)	<0.001
CAD, n (%)	196,030 (54.9)	887 (9.5)	<0.001
HF, n (%)	75,334 (21.1)	1,304 (14.0)	<0.001
Prior stroke, n (%)	85,302 (23.9)	1,506 (16.1)	<0.001
Prior MI, n (%)	44,406 (12.4)	2,862 (30.7)	<0.001
Prior revascularization, n (%)	81,584 (22.8)	3,481 (37.3)	<0.001
CKD stage, n (%)			<0.001
Stage 1	57,932 (16.2)	3,700 (39.6)	
Stage 2	142,760 (40.0)	3,656 (39.2)	
Stage 3 or 4	156,383 (43.8)	1,980 (21.2)	
Any cardiovascular disease, n (%)	303,221 (84.9)	7,731 (82.8)	<0.001
Hypertension, n (%)	306,579 (85.9)	8,508 (91.1)	<0.001
Hyperlipidemia, n (%)	246,848 (69.1)	7,067 (75.7)	<0.001
Atrial fibrillation, n (%)	58,036 (16.3)	179 (1.9)	<0.001
Dementia, n (%)	19,146 (5.4)	16 (0.2)	<0.001
COPD, n (%)	79,735 (22.3)	135 (1.4)	<0.001
Cancer, n (%)	131,325 (36.8)	551 (5.9)	<0.001
Liver disease, n (%)	26,929 (7.5)	594 (6.4)	<0.001
Smoking status, n (%)			<0.001
Current	77,955 (21.8)	1,130 (12.1)	
Former	199,577 (55.9)	4,338 (46.5)	
Never	79,543 (22.3)	3,868 (41.4)	

Abbreviations: BMI, Body mass index; eGFR, estimated glomerular filtration rate; CAD, coronary artery disease; HF, heart failure; MI, myocardial infarction; CKD, chronic kidney disease; COPD, chronic obstructive pulmonary disease

## Data Availability

Code for all statistical analysis is available upon request. A deidentified, anonymized limited data set derived from the datasets used for the analysis can be made available upon reasonable request from researchers with necessary human subjects research oversight and in accordance with VA data sharing policies.

## What is already known on this topic:

- The LEADER trial demonstrated that liraglutide, a GLP-1 receptor agonist, reduces cardiovascular events in patients with type 2 diabetes, but the trial enrolled few US veterans who have higher cardiovascular risk than the general diabetes population.
- While GLP-1 receptor agonists have proven cardiovascular benefits in clinical trials, their effectiveness in real-world populations with different characteristics than trial participants are unclear, particularly for healthcare systems with patients who are underrepresented in clinical trials.

## What this study adds:

- Our analysis shows that liraglutide’s cardiovascular benefits observed in LEADER generalize to US veterans with type 2 diabetes, with potentially greater absolute risk reductions for major cardiovascular events and mortality in this high-risk population.
- We demonstrate how to extend trial results to a real-world population that may be underrepresented in the trial sample using methods that preserve randomization benefits while accounting for population differences.
- Results suggest that current VA formulary restrictions based on trial inclusion criteria (prior cardiovascular disease, elevated HbA1c) may unnecessarily limit access to beneficial therapy, as cardiovascular benefits were consistent across a more broadly defined veteran population.
