# Supplementary material for: Real-world cardiovascular effects of liraglutide: transportability analysis of the LEADER trial
Source: medRxiv. 2025 May 30:2025.05.12.25327466. Preprint. [Version 2] doi: 10.1101/2025.05.12.25327466 (PMC12132110; doi:10.1101/2025.05.12.25327466)
Supplement: Supplement 1 [file media-1.pdf]

Real-world cardiovascular effects of liraglutide: transportability analysis of the LEADER trial

<u>Online-only Supplemental Material</u>

Table of Contents

<u>Supplemental Methods</u>

The Purpose of Transporting Experimental Results

Pseudo-observations for Survival Probabilities

Identification of the Transported Average Treatment Effect

Approximate Balancing Weights for Doubly-Robust Transport of Treatment Effects

References

<u>Supplemental Results</u>

eTable 1. Inclusion/exclusion criteria for LEADER study and mapping to VA data.

eTable 2. Baseline participant characteristics in LEADER and across VA target populations.

eFigure 1. Covariate balance plots comparing LEADER to VA target populations before and after application of approximate balancing weights.

eFigure 2. Variation in transported effect estimates without and with sex as a balancing variable.

eFigure 3. Leave-one-out analysis examining influence of single balancing variables on transported effect estimates.

eFigure 4. Sensitivity of transported hazard ratio estimates to varying VA target population definitions.

eFigure 5. Effective sample sizes when transporting LEADER to nested VA target populations.

Supplemental Methods

The Purpose of Transporting Experimental Results

Transporting effect estimates from a randomized controlled trial (RCT), such as the LEADER trial evaluating liraglutide's effects on major adverse cardiovascular events (MACE), to a broader observational cohort like the Veteran Affairs (VA) population of type II diabetic patients is critically important. This approach ensures findings are relevant and applicable to real-world populations, which often differ significantly in demographic, clinical, and treatment characteristics from a typically highly selected trial cohort. Specifically, the VA population may exhibit unique comorbidity patterns, demographic and socioeconomic influences, or healthcare access disparities not fully represented by the findings of a clinical trial.

Several methodological strategies are commonly employed for this effect transportation[1,2]:

1. **Weighting Methods (Inverse Probability Weighting - IPW):** Adjusts for differences between the trial and target (VA) populations by assigning weights based on the likelihood of trial participation given observed covariates. This reweights the RCT sample to resemble the VA cohort, enabling generalizable estimates.
2. **G-Computation:** Involves modeling the outcome as a function of treatment and covariates to predict outcomes under different treatment scenarios in the target population.
3. **Doubly Robust (DR) Methods (e.g., Augmented IPW - AIPW):** Combines weighting and outcome modeling, achieving robustness by requiring only one of these two models (propensity for trial inclusion or outcome regression) to be correctly specified.

Applying these methods to transport the LEADER trial results specifically to the VA diabetic population allows policymakers and clinicians to make more informed decisions regarding liraglutide's cardiovascular benefits within this distinct and medically complex patient population. We focus on the latter of the three methods as: 1) it combines the other two methods;[3] and 2) it enables the operationalization of machine learning to provide more robust, data-driven estimates without sacrificing parametric rates of convergence[4] (e.g. the central-limit theorem holds for DR estimators despite using models that do not necessarily confer root-n consistency).

Pseudo-observations for Survival Probabilities

Pseudo-observations, as introduced by Per Kragh Andersen and Pohar Perme[5], provide a method for estimating survival probabilities at a given time using a leave-one-out jackknife approach. This technique is particularly useful in regression modeling where direct estimation of individual survival probabilities may be challenging.

For participant $i = 1,2, \ldots, n$, and given the trial data, where the samples have survival data, let be the Kaplan-Meier estimator of the survival function at time t. The individual pseudo-observations are defined as:

$$\tilde{S}_i(t) = \hat{S}(t) - \hat{S}^{(-i)}(t)$$

where $\hat{S}(t)$ is the Kaplan-Meier estimate of survival at time using all data at time t, and $\hat{S}^{(-i)}(t)$ is the Kaplan-Meier estimate of survival at time t computed after removing individual from the dataset.

Identification of the Transported Average Treatment Effect

Starting with the notation from the previous section, let $Z_i \in \{0,1\}$ indicate whether a sample belongs to the trial ($Z_i = 1$) or to the target ($Z_i = 0$), $A_i \in \{0,1\}$ be the indicator for treatment with Liraglutide, and expand the definition of the pseudo-observations with $S_i(t,1)$ and $S_i(t,0)$ representing the potential survival probability pseudo-outcomes under treatment ($A_i = 1$) and control ($A_i = 0$), respectively. Finally Define $\boldsymbol{X}_i = \left(1, X_{i1}, X_{i2}, \dots, X_{ip}\right)^T$ as the set of covariates that we will need to balance and condition or outcome regression upon. The parameter of interest is the target population average treatment effect on survival, which is defined as

$$\theta_0(t) = E[S_i(t,1) - S_i(t,0) | Z_i = 0].$$

To be able to estimate this quantity with the observed data (noting that half of the potential outcomes, fundamentally, are unobserved) requires a few assumptions:

1. <u>*Stable Unit Treatment Value Assumption (SUTVA):*</u> consists of two conditions:
    - *No interference*: The exposure assignment one person does not influence the potential outcome of others;
    - *Consistency:* The observed outcome corresponds with the factual potential outcome.
2. <u>*Exposure Ignorability:*</u> $S_i(t,a) \perp A_i | \boldsymbol{X}_i, Z_i = 1$
    - The potential outcomes are independent of exposure when we condition on the confounders (covariates that influence both the outcome and exposures) – specifically in the trial sample ($Z_i = 1$).
    - We assume that $\boldsymbol{X}_i$ is sufficient to capture confounding.
3. <u>*Sample Exchangeability*</u>*:* The average of the difference between potential outcomes is exchangeable across the trial and the target sample:

$$E[S_i(t,1) - S_i(t,0) | \boldsymbol{X}_i, Z_i = 0] = E[S_i(t,1) - S_i(t,0) | \boldsymbol{X}_i, Z_i = 1]$$

4) <u>*Positivity*</u>: For the sample indicator: $0 < \Pr\{Z_i = 1 | \boldsymbol{X}_i\} < 1$.

We can then express the average treatment effect of the potential outcome as

$$
\begin{aligned}
\theta_0(t) &= E_S[S_i(t,1) - S_i(t,0)|Z_i = 0] \\
&= E_X\{E_S[S_i(t,1) - S_i(t,0)|\boldsymbol{X}_i]|Z_i = 0\} && \text{(Iterative Expectation)} \\
&= E_X\{E_S[S_i(t,1) - S_i(t,0)|\boldsymbol{X}_i, Z_i = 1]|Z_i = 0\} && \text{(Exchangeability)} \\
&= E_X\{E_S[S_i(t,1)|A_i = 1, \boldsymbol{X}_i, Z_i = 1]|Z_i = 0\} \\
&\quad - E_X\{E_S[S_i(t,0)|A_i = 0, \boldsymbol{X}_i, Z_i = 1]|Z_i = 0\} && \text{(Ignorability)} \\
&= E_X\{E_S[S_i(t)|A_i = 1, \boldsymbol{X}_i, Z_i = 1]|Z_i = 0\} \\
&\quad - E_X\{E_S[S_i(t)|A_i = 0, \boldsymbol{X}_i, Z_i = 1]|Z_i = 0\} && \text{(Consistency)}
\end{aligned}
$$

where the latter expression is composed entirely of observable data.

(Approximate) Balancing Weights for Doubly Robust Transport of Treatment Effects

In observational studies and causal inference, balancing weights are frequently used to reweight samples for unbiased estimation of treatment effects. Approximate balancing weights extend these methods by directly optimizing for covariate balance while maintaining statistical efficiency. When applied in a doubly robust framework, these weights enable the transportability of treatment effects across different populations with several theoretical advantages.

The estimator we will implement to infer the target population average treatment effects requires some estimated weights $\hat{\gamma}_i$, $i = 1,2,\ldots,n$, which are constructed to modify the marginal distribution of covariates $\boldsymbol{X}_i$, particularly the distribution of any effect modifiers, so that the weighted covariate means in the trial sample ($Z_i = 1$) match those of the target population represented by a target sample ($Z_i = 0$). This can be formalized as the following optimization problem that identifies the weights that balance the covariate moments within a standardized range[6] δ_j, $j = 1,2,\ldots,p$:

$$
\min_{\boldsymbol{\gamma} \in \Re^n} \sum_{i=1}^{n} Z_i(\gamma_i \log \gamma_i - \gamma_i)
$$

$$
\text{subject to } \left| \sum_{i=1}^{n} \gamma_i Z_i X_{ij} - \sum_{i=1}^{n} (1 - Z_i) X_{ij} \right| \leq \delta_j \text{ for all } j = 1,2,\ldots,p
$$

This objective function is known as the primal problem.[7] So long as the X_{ij} are linearly independent, the primal problem is equivalent to optimizing a Lagrangian dual that finds

$$
\hat{\boldsymbol{\lambda}} = \underset{\boldsymbol{\lambda} \in \Re^p}{\operatorname{argmin}} \sum_{i=1}^{n} \left[\exp\left(-Z_i \sum_{j=1}^{p} X_{ij}\lambda_j \right) + \sum_{j=1}^{p} (1 - Z_i) X_{ij}\lambda_j \right] + \sum_{j=1}^{p} \delta_j |\lambda_j|
$$

where the connection between the dual solution $\hat{\boldsymbol{\lambda}} = \left(\hat{\lambda}_1, \hat{\lambda}_2, \ldots, \hat{\lambda}_p\right)^T$ and the primal solutions is

$$\hat{\gamma}_\mathrm{i} = \exp\left(-\boldsymbol{X}_i^T \hat{\boldsymbol{\lambda}}\right).$$

When $\delta_j = 0$ for all $j = 1,2, \ldots, p$, then we obtain the entropy balancing weights. However, if we select $\delta_j = c\sigma_j$ where σ_j is the standard deviation of X_{ij} in the target population, then we are in effect restricting the absolute standardized mean difference between the weighted trial covariates and the covariates in the target sample to fall within a constant range c of zero. This formulation ensures that the weighted distribution of covariates closely approximates that of the target population while controlling possible variance inflation, which can be detected through the effective sample size

$$ESS = \frac{(\sum_{i=1}^{n} Z_i \hat{\gamma}_i)^2}{\sum_{i=1}^{n} Z_i \hat{\gamma}_i{}^2}$$

We set $c = 0.05$ to demonstrate the improved effective sample size achievable by introducing the potential bias. Note that it is generally recommended that $X_{i1} = 1$ for all $i = 1,2, \ldots, n$ such that $\sum_i \hat{\gamma}_i Z_i = \sum_i (1 - Z_i) = n_0$ with $\delta_1 = 0$, conferring exact balance of the sample size.

Also, observe that these weights do not make any attempt to balance differences between treatment groups, despite seeing more precise effect estimates by also balancing treatment. We opted instead for simplicity, relying on the randomization of treatment in the trial sample, implying we should still obtain consistent and asymptotically efficient effect estimates.

The doubly robust augmented inverse probability weighted estimator combines both outcome modeling and weighting to correct for potential model misspecifications.[1,3] The estimated expected treatment effect in the target population ($Z_i = 0$) at time t is:

$$\hat{\theta}_0(t) = \frac{1}{n_0} \sum_{i=1}^{n} \hat{\psi}_0[A_i, \boldsymbol{X}_i, Z_i, S_i(t)]$$

where

$$\hat{\psi}_0[A_i, \boldsymbol{X}_i, Z_i, S_i(t)] = Z_i \hat{\gamma}_\mathrm{i}[S_i(t) - \hat{\mu}_t(A_i, \boldsymbol{X}_i)] + (1 - Z_i)[\hat{\mu}_t(A_i = 1, \boldsymbol{X}_i) - \hat{\mu}_t(A_i = 0, \boldsymbol{X}_i)].$$

The balancing weights $\hat{\gamma}_i$ are fit using the Lagrangian dual optimization problem from earlier. As mentioned in the main manuscript, we use SuperLearner[8] to fit the outcome model $\hat{\mu}_t(A_i, \boldsymbol{X}_i) = \hat{E}[S_i(t) | A_i, \boldsymbol{X}_i, Z_i = 1]$ with an ensemble of prediction algorithms incorporating generalized linear model regressions, elastic net regressions, multivariate adaptive spline regressions, and random forest regressions. Note that $S_i(t)$ is not observed, so we use $\tilde{S}_i(t)$ instead.

By combining the weighting method with an outcome model, this approach remains consistent even if one of the two components (weights or outcome model) is misspecified. In addition, the combination of both estimators ensures that if both parameters converge

at a slower-than-parametric rate of convergence, we will still get consistent effect estimates.

From a methodological standpoint, this study demonstrates the feasibility of using doubly robust estimators and balancing weights in a complex survival context. We showed that by using approximate balancing, we achieved excellent covariate balance with a much larger effective sample size than traditional weighting (which often would down-weight most trial patients heavily if they differed greatly from the target). This technique may be useful in other transportability scenarios where exact balance is difficult or leads to inefficiency.

References

1. Dahabreh IJ, Robertson SE, Steingrimsson JA, Stuart EA, Hernan MA. Extending inferences from a randomized trial to a new target population. *Stat Med.* 2020;39(14):1999-2014.
2. Degtiar I, Rose S. A Review of Generalizability and Transportability. *Annual Review of Statistics and Its Application.* 2023;10(1):null.
3. Lee D, Yang S, Dong L, Wang X, Zeng D, Cai J. Improving trial generalizability using observational studies. *Biometrics.* 2023;79(2):1213-1225.
4. Chernozhukov V, Chetverikov D, Demirer M, et al. Double/debiased machine learning for treatment and structural parameters. *The Econometrics Journal.* 2018;21(1):C1-C68.
5. Andersen PK, Perme MP. Pseudo-observations in survival analysis. *Stat Methods Med Res.* 2010;19(1):71-99.
6. Keele LJ, Ben-Michael E, Feller A, Kelz R, Miratrix L. Hospital quality risk standardization via approximate balancing weights. *The Annals of Applied Statistics.* 2023;17(2):901-928, 928.
7. Josey KP, Juarez-Colunga E, Yang F, Ghosh D. A framework for covariate balance using Bregman distances. *Scandinavian Journal of Statistics.* 2021;48(3):790-816.
8. van der Laan MJ, Polley EC, Hubbard AE. Super Learner. *Statistical Applications in Genetics and Molecular Biology.* 2007;6(1).

Supplemental Results

eTable 1. Inclusion/Exclusion criteria of the LEADER study and mapping to VA data.

**LEADER**	**VA**
**Baseline date**	**Baseline date**
Date of randomization	Earliest date after meeting all below inclusion/exclusion criteria that any new diabetes medication prescription was filled in VA care
**Major inclusion criteria**	**Major inclusion criteria**
Patients with type 2 diabetes	Patients with type 2 diabetes based on ICD 9 or 10 codes[a]
Treatment history:<br><br>i. No diabetes medications<br>ii. One or more oral antihyperglycemic agents<br>iii. Selected insulins (human neutral protamine Hagedorn, long-acting analogue, premixed)<br>iv. Combination of above	Treatment history from VA pharmacy records and linked data from CMS data:<br>i. No diabetes medications<br>ii. One or more oral antihyperglycemic agents<br>iii. Selected insulins (human neutral protamine Hagedorn, long-acting analogue, premixed)<br>iv. Combination of above
HbA1c ≥ 7.0%	HbA1c ≥ 7.0%
Age ≥ 50 years with at least one cardiovascular coexisting condition:	Age ≥ 50 years (from VA enrollment records) with at least one cardiovascular coexisting condition[b,c]:
i. Coronary heart disease	i. ICD9 or 10 codes or procedure codes for myocardial infarction, coronary artery disease, percutaneous coronary intervention, coronary artery bypass graft
ii. Cerebrovascular disease	ii. ICD9 or 10 codes for stroke, transient ischemic attack, cerebrovascular disease
iii. Peripheral vascular disease	iii. ICD9 or 10 codes or procedure codes for peripheral vascular disease
iv. Chronic kidney disease of stage 3 or greater	iv. Baseline CKD stage 3 or greater based on eGFR <60 ml/min/1.73m^2 calculated using MDRD equation<br>OR<br>ICD9 or 10 codes for chronic renal failure
v. Chronic heart failure of New York Heart Association class II or III	v. ICD9 or 10 codes for congestive heart failure, systolic heart failure

Age ≥ 60 years with at least one cardiovascular risk factor, as determined by the investigator: Microalbuminuria or proteinuria; hypertension and left ventricular hypertrophy, left ventricular systolic or diastolic dysfunction, or an ankle-brachial index of less than 0.9.	Not mapped due to poor availability of standardized high-quality data related to albuminuria, echocardiographic parameters, ankle brachial index
**Major exclusion criteria**	**Major exclusion criteria**
Type 1 diabetes	Type 1 diabetes based on ICD9 or 10 codes
Use of GLP-1 receptor agonists, dipeptidyl peptidase 4 (DPP-4) inhibitors, pramlintide, or rapid-acting insulin	Treatment history from VA pharmacy records to exclude individuals based on VA prescription for GLP-1RA, DPP-4 inhibitors, pramlintide, or rapid-acting insulin
Familial or personal history of multiple endocrine neoplasia type 2 or medullary thyroid cancer	Not mapped
Occurrence of an acute coronary or cerebrovascular event within 14 days before screening and randomization	No inpatient or outpatient ICD9 or 10 diagnosis codes for myocardial infarction, stroke, transient ischemic attack
	eGFR <15 ml/min/1.73m^2 or on dialysis<br>• Additional exclusion criterion due to low numbers in among otherwise eligible individuals and uncertain VA eligibility for GLP-1RA treatment

[a] Safford et al. *Diabetes Care*. 2004; 27 Suppl 2: B10-21.

[b] Raghavan et al. *Journal of the American Heart Association*. 2019; 8(4): e011295.

[c] Honerlaw et al. *Journal of the American Medical Informatics Association*. 2024; 31(5): 1126-1134.

eTable 2. Baseline participant characteristics in LEADER and across VA target populations.

	LEADER	VA A	p-value	VA B	p-value	VA C	p-value	VA D	p-value	VA E	p-value
n	9,336	357,075		494,682		370,536		568,311		893,420	
Male sex, n (%)	6,001 (64.3)	348,101 (97.5)	<0.001	481,991 (97.4)	<0.001	360,526 (97.3)	<0.001	550,033 (96.8)	<0.001	857,093 (95.9)	<0.001
Race, n (%)			<0.001		<0.001		<0.001		<0.001		<0.001
Black or African American	776 (8.3)	50,312 (14.1)		71,031 (14.4)		53,832 (14.5)		95,806 (16.9)		163,913 (18.3)	
White	7,237 (77.5)	279,341 (78.2)		385,283 (77.9)		287,939 (77.7)		47,095 (8.3)		78,106 (8.7)	
Other	1,323 (14.2)	27,422 (7.7)		38,368 (7.8)		28,765 (7.8)		425,410 (74.9)		651,401 (72.9)	
Age (years), mean (SD)	64.3 (7.2)	70.0 (8.7)	<0.001	70.3 (8.7)	<0.001	69.1 (9.8)	<0.001	68.0 (8.9)	<0.001	65.7 (11.5)	<0.001
Hemoglobin A1c (%), mean (SD)	8.7 (1.5)	8.8 (1.7)	<0.001	8.1 (1.8)	<0.001	8.8 (1.7)	<0.001	8.8 (1.7)	<0.001	8.2 (1.9)	<0.001
# of diabetes medications, n (%)			<0.001		<0.001		<0.001		<0.001		<0.001
≤1	1,313 (14.1)	173,632 (48.6)		281,613 (56.9)		183,189 (49.4)		312,449 (55.0)		585,377 (65.5)	
2	3,294 (35.3)	87,554 (24.5)		103,695 (21.0)		89,937 (24.3)		129,557 (22.8)		160,262 (17.9)	
≥3	4,729 (50.7)	95,889 (26.9)		109,374 (22.1)		97,410 (26.3)		126,305 (22.2)		147,781 (16.5)	
BMI (kg/m^2), mean (SD)	32.5 (6.3)	32.6 (6.4)	0.466	32.4 (6.4)	0.215	32.7 (6.5)	0.011	32.7 (6.4)	0.021	32.9 (6.5)	<0.001
eGFR (ml/min/1.73m^2), mean (SD)	79.1 (22.1)	66.4 (21.4)	<0.001	66.1 (21.2)	<0.001	67.4 (22.0)	<0.001	73.4 (21.0)	<0.001	75.7 (21.9)	<0.001
CAD, n (%)	887 (9.5)	196,030 (54.9)	<0.001	271,445 (54.9)	<0.001	201,690 (54.4)	<0.001	196,030 (34.5)	<0.001	279,045 (31.2)	<0.001
HF, n (%)	1,304 (14.0)	75,334 (21.1)	<0.001	105,025 (21.2)	<0.001	77,450 (20.9)	<0.001	75,334 (13.3)	0.046	107,882 (12.1)	<0.001
Prior stroke, n (%)	1,506 (16.1)	85,302 (23.9)	<0.001	116,486 (23.5)	<0.001	87,758 (23.7)	<0.001	85,302 (15.0)	0.003	119,814 (13.4)	<0.001
Prior MI, n (%)	2,862 (30.7)	44,406 (12.4)	<0.001	60,408 (12.2)	<0.001	46,177 (12.5)	<0.001	44,406 (7.8)	<0.001	62,788 (7.0)	<0.001

Prior revascularization, n (%)	3,481 (37.3)	81,584 (22.8)	<0.001	110,898 (22.4)	<0.001	83,162 (22.4)	<0.001	81,584 (14.4)	<0.001	112,934 (12.6)	<0.001
CKD stage, n (%)			<0.001		<0.001		<0.001		<0.001		<0.001
Stage 1	3,700 (39.6)	57,932 (16.2)		76,367 (15.4)		66,076 (17.8)		137,732 (24.2)		249,322 (27.9)	
Stage 2	3,656 (39.2)	142,760 (40.0)		199,849 (40.4)		146,353 (39.5)		274,196 (48.2)		423,287 (47.4)	
Stage 3 or 4	1,980 (21.2)	156,383 (43.8)		218,466 (44.2)		158,107 (42.7)		156,383 (27.5)		220,811 (24.7)	
Any cardiovascular disease, n (%)	7,731 (82.8)	303,221 (84.9)	<0.001	416,681 (84.2)	<0.001	315,641 (85.2)	<0.001	303,221 (53.4)	<0.001	433,270 (48.5)	<0.001
Hypertension, n (%)	8,508 (91.1)	306,579 (85.9)	<0.001	424,287 (85.8)	<0.001	315,309 (85.1)	<0.001	478,024 (84.1)	<0.001	727,632 (81.4)	<0.001
Hyperlipidemia, n (%)	7,067 (75.7)	246,848 (69.1)	<0.001	339,547 (68.6)	<0.001	254,379 (68.7)	<0.001	383,962 (67.6)	<0.001	586,671 (65.7)	<0.001
Atrial fibrillation, n (%)	179 (1.9)	58,036 (16.3)	<0.001	84,367 (17.1)	<0.001	58,502 (15.8)	<0.001	67,460 (11.9)	<0.001	100,482 (11.2)	<0.001
Dementia, n (%)	16 (0.2)	19,146 (5.4)	<0.001	27,031 (5.5)	<0.001	19,224 (5.2)	<0.001	22,900 (4.0)	<0.001	33,080 (3.7)	<0.001
COPD, n (%)	135 (1.4)	79,735 (22.3)	<0.001	113,715 (23.0)	<0.001	80,846 (21.8)	<0.001	102,705 (18.1)	<0.001	152,681 (17.1)	<0.001
Cancer, n (%)	551 (5.9)	131,325 (36.8)	<0.001	182,061 (36.8)	<0.001	133,405 (36.0)	<0.001	198,533 (34.9)	<0.001	293,270 (32.8)	<0.001
Liver disease, n (%)	594 (6.4)	26,929 (7.5)	<0.001	37,403 (7.6)	<0.001	28,415 (7.7)	<0.001	43,101 (7.6)	<0.001	71,164 (8.0)	<0.001
Smoking status, n (%)			<0.001		<0.001		<0.001		<0.001		<0.001
Current	1,130 (12.1)	77,955 (21.8)		106,125 (21.5)		82,878 (22.4)		122,362 (21.5)		195,686 (21.9)	
Former	4,338 (46.5)	199,577 (55.9)		280,102 (56.6)		204,111 (55.1)		310,955 (54.7)		480,631 (53.8)	
Never	3,868 (41.4)	79,543 (22.3)		108,455 (21.9)		83,547 (22.5)		134,994 (23.8)		217,103 (24.3)	

Abbreviations: BMI, Body mass index; eGFR, estimated glomerular filtration rate; CAD, coronary artery disease; HF, heart failure; MI, myocardial infarction; CKD, chronic kidney disease; COPD, chronic obstructive pulmonary disease

eFigure 1. Covariate balance plots comparing LEADER to VA target populations before and after application of approximate balancing weights.

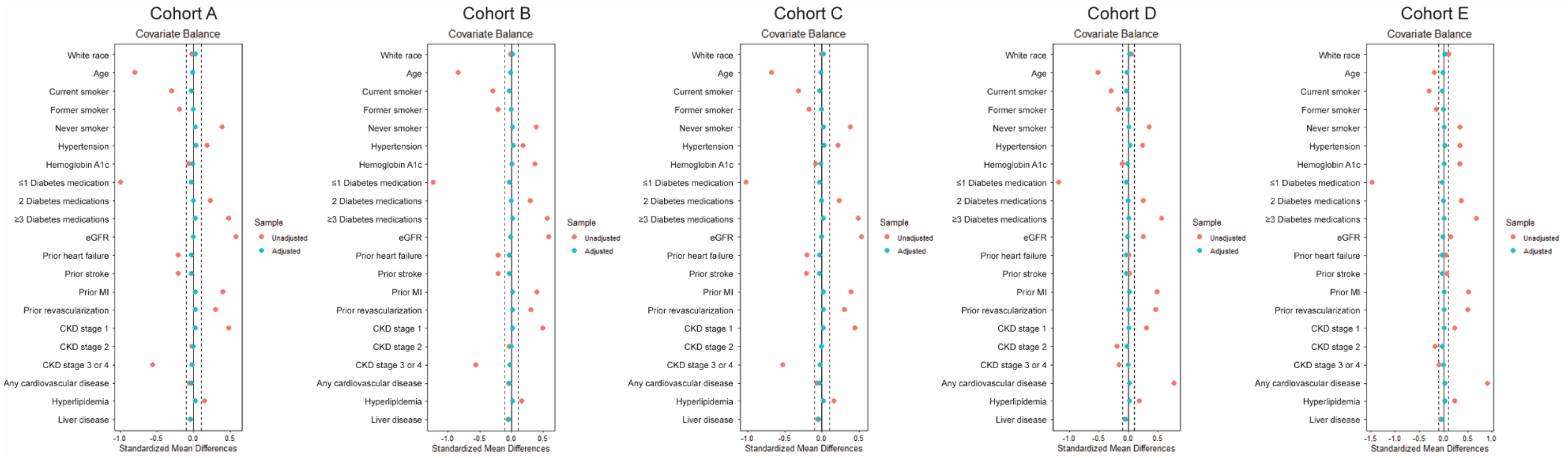

eFigure 1. Standardized mean differences (SMD) for baseline covariates before (blue markers) and after (red markers) weighting in each VA cohort (A-E) using approximate balancing weights. The vertical dashed lines (at ±0.1) denote the threshold for negligible imbalance. Baseline differences evident between the LEADER and VA populations are mitigated substantially reduced after weighting.

eFigure 2. Variation in transported effect estimates without and with sex as a balancing variable.

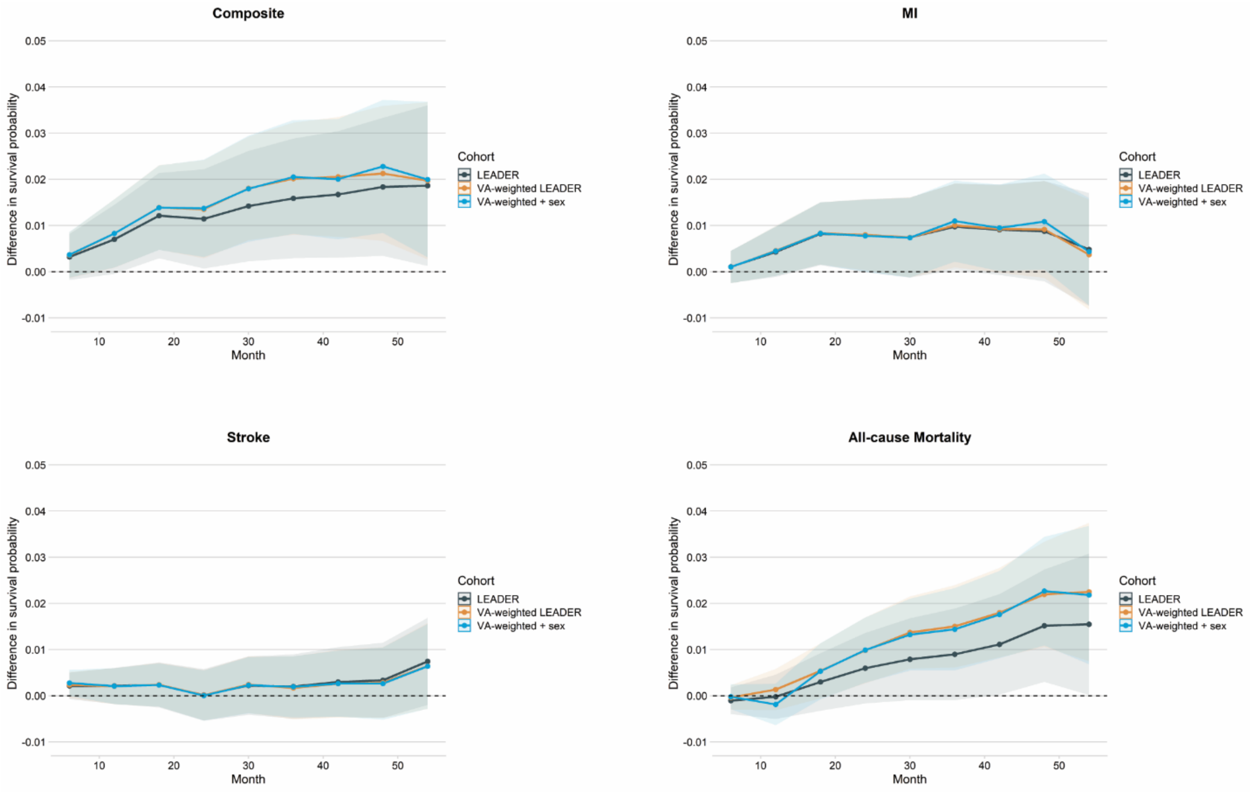

eFigure 2. Comparison of treatment effects estimated as survival probability differences for MACE, non-fatal MI, non-fatal stroke, and all-cause mortality in LEADER (dark blue), LEADER transported to the primary VA target population without including sex as a balancing variable *(*yellow), and LEADER transported to the primary VA target population including sex as a balancing variable (light blue).

eFigure 3. Leave-one-out analysis examining influence of single balancing variables on transported effect estimates.

(A)

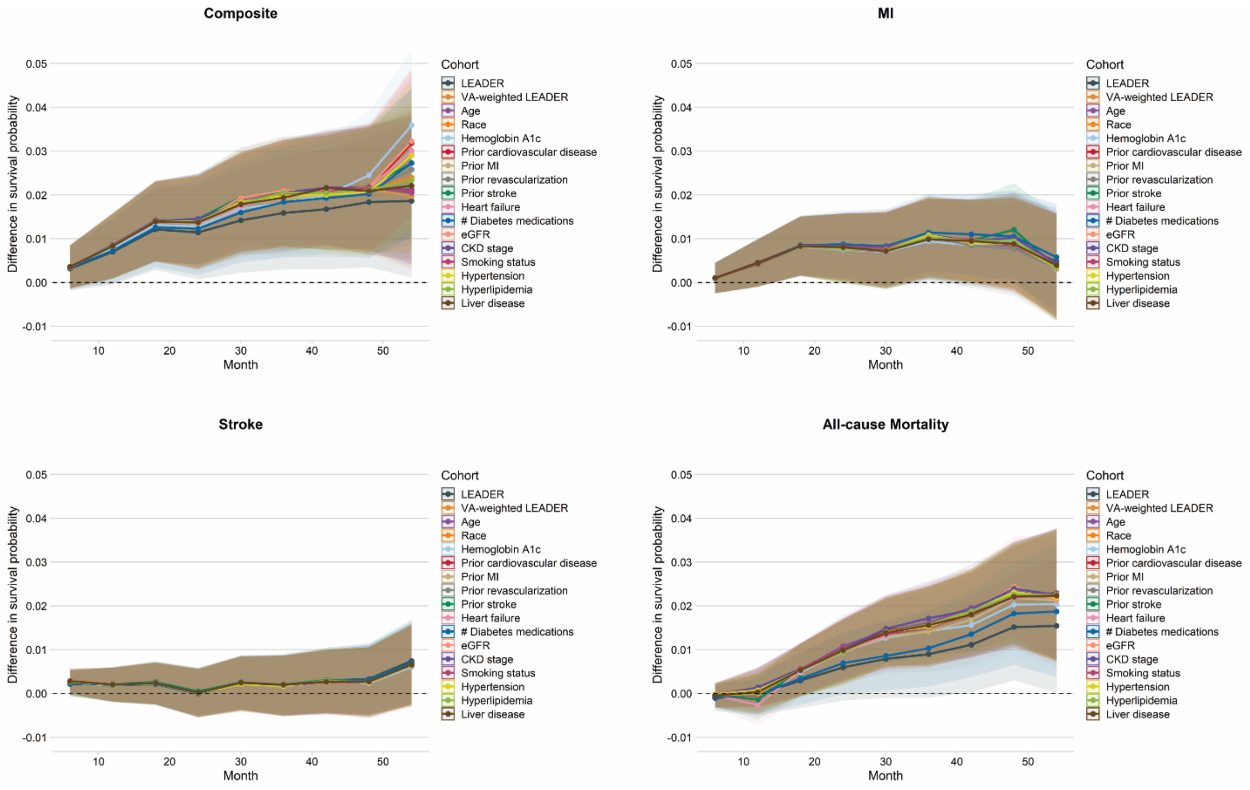

(B)

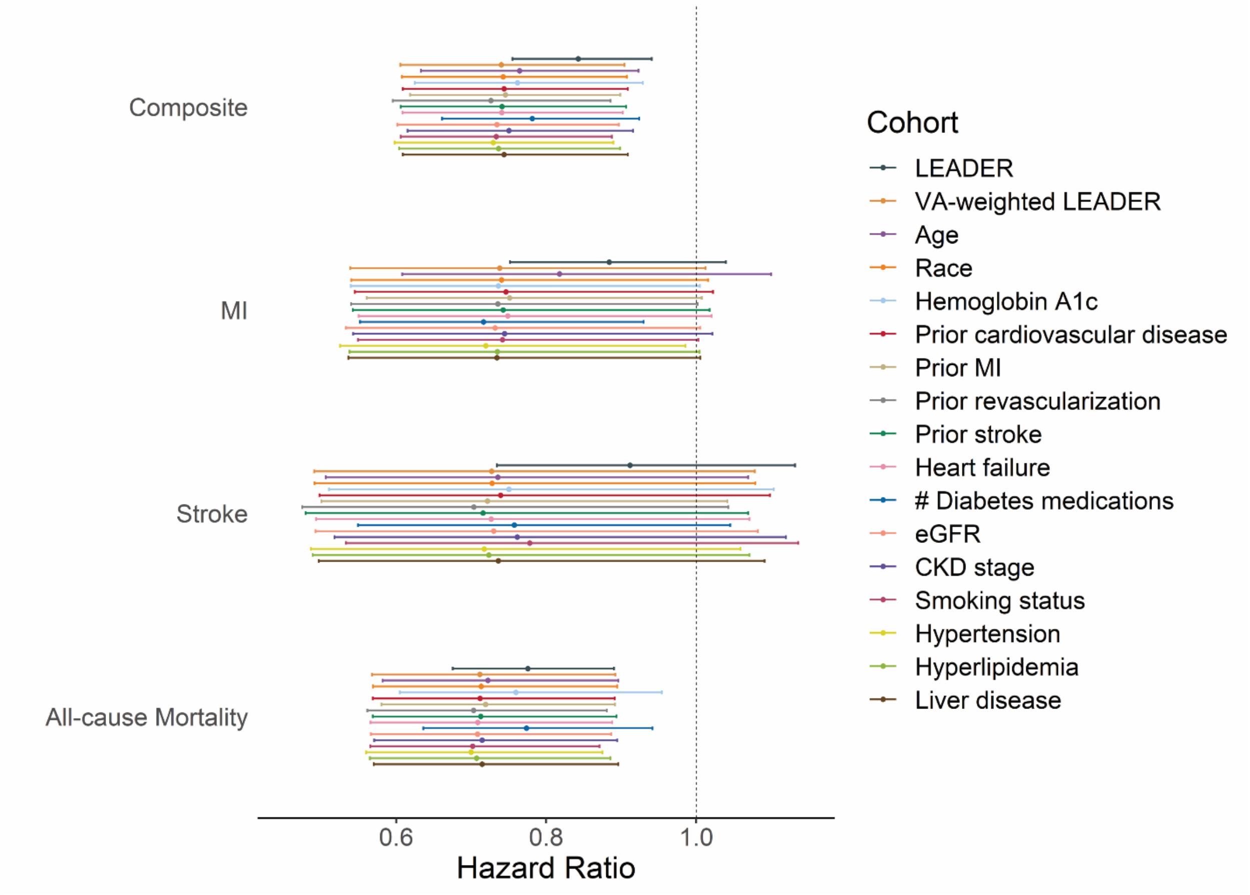

eFigure 3. (A) Comparison of treatment effects in LEADER and LEADER transported to the VA target population, varying the set of balancing covariates by leaving out one covariate at a time. Each line represents a transported treatment effect estimated as survival probability differences (A) or HR (B) for MACE, non-fatal MI, non-fatal stroke, and all-cause mortality when balancing with all variables used in the primary analyses except the variable indicated in the legend. 95% Confidence intervals are shown with colored sharing for each transported treatment effect estimate (A) or as error bars (B).

eFigure 4. Sensitivity of transported hazard ratio estimates to varying VA target population definitions.

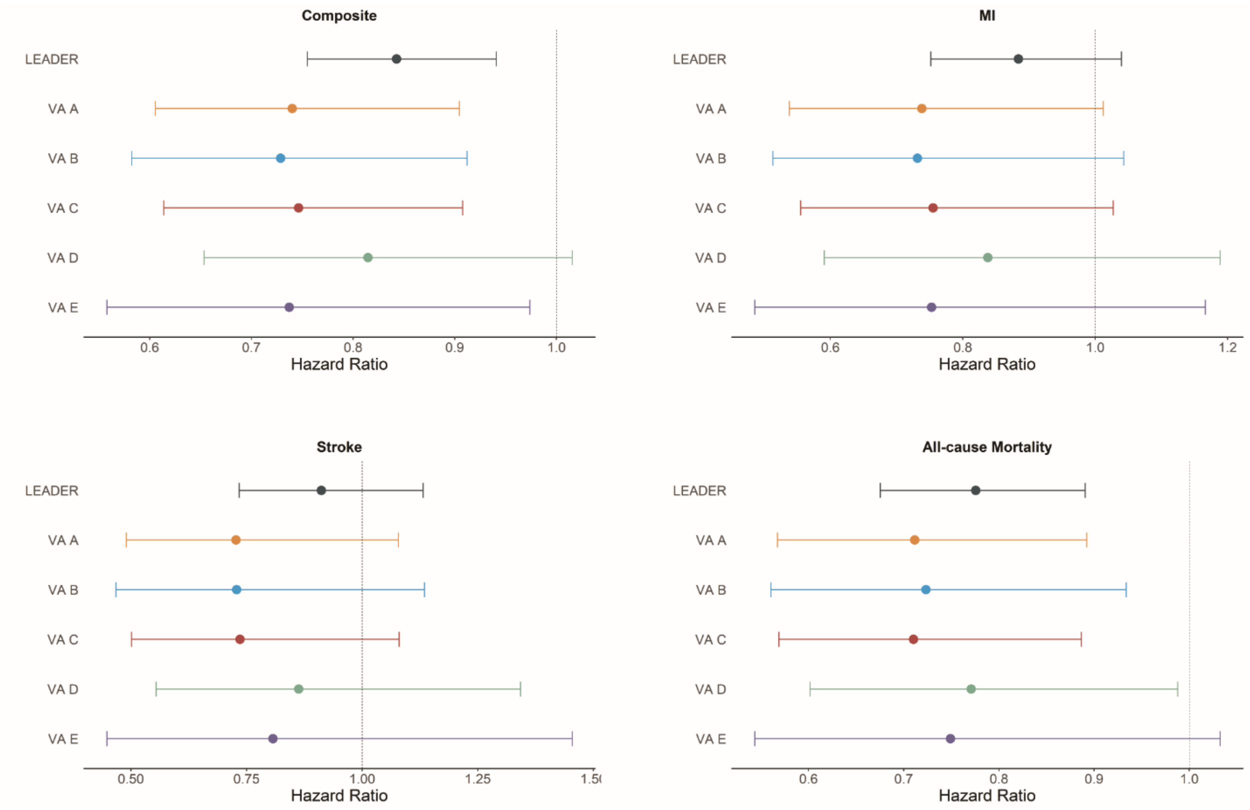

eFigure 4. Comparison of treatment effects in LEADER and LEADER transported to series of VA target populations with varying inclusion/exclusion criteria. Transported treatment effects estimated as hazard ratios (HR) for composite major adverse cardiovascular events (Composite), non-fatal myocardial infarction (MI), non-fatal stroke, and all-cause mortality; 95% confidence intervals are shown as error bars. VA A through VA E indicate results after weighting LEADER to the corresponding target population.

eFigure 5. Effective sample sizes when transporting LEADER to nested VA target populations.

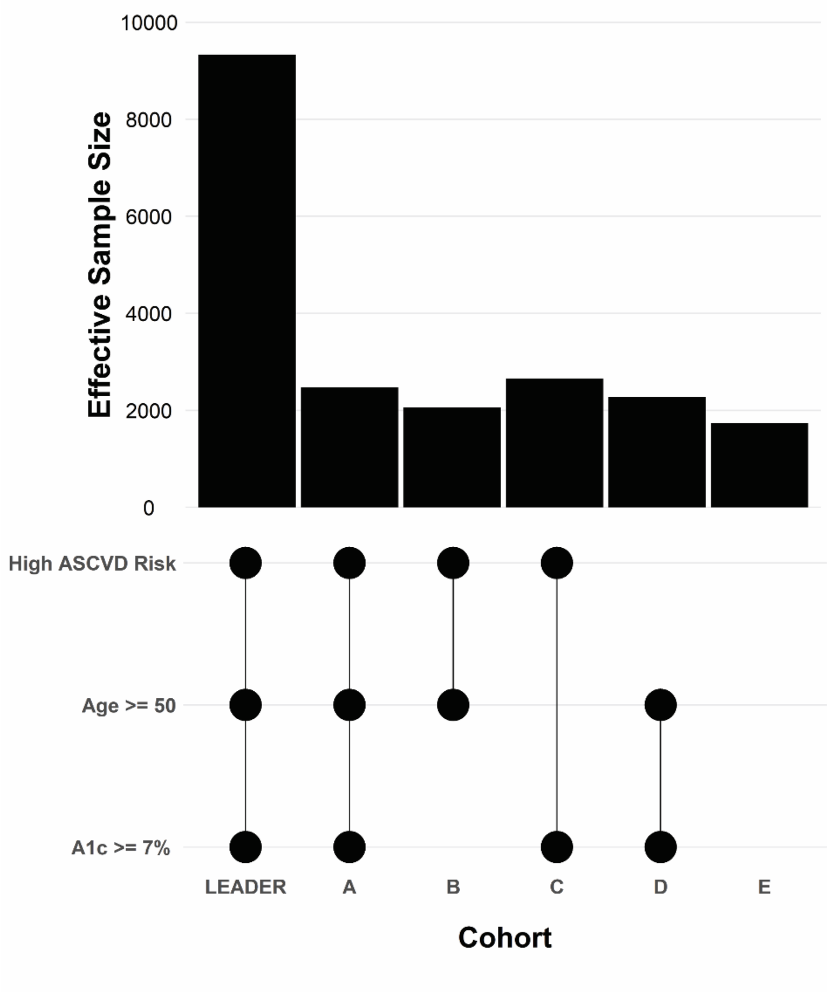

eFigure 5. Comparison of key inclusion criteria from LEADER and definition of different VA target populations, labeled Cohorts A-E (lower), and effective sample sizes when weighting LEADER data to transport to each Cohort (above).
